# LEDA—Layered Event-Based Malware Detection Architecture

**DOI:** 10.3390/s24196393

**Published:** 2024-10-02

**Authors:** Radu Marian Portase, Raluca Laura Portase, Adrian Colesa, Gheorghe Sebestyen

**Affiliations:** 1 Computer Science Department, Technical University of Cluj Napoca, 400114 Cluj Napoca, Romania; 2 Bitdefender, 060071 Bucharest, Romania

**Keywords:** real-time malware detection, process behavior monitoring, machine learning, malware detection architecture

## Abstract

The rapid increase in new malware necessitates effective detection methods. While machine learning techniques have shown promise for malware detection, most research focuses on identifying malware through the content of executable files or full behavior logs collected from process start to finish. However, detecting threats like ransomware via full logs is redundant, as this malware type openly informs users of the infection. To address this, we present LEDA, a novel malware detection architecture designed to monitor process behavior during execution and to identify malicious actions in real time. LEDA dynamically learns the most relevant features for detection and optimally triggers model evaluations to minimize the performance impact perceived by users. We evaluated LEDA using a dataset of Windows malware and legitimate applications collected over a year, examining our model’s temporal decay in effectiveness.

## 1. Introduction

Detecting malware, also known as malicious software, is one of the greatest challenges of the 21st century [1]. To perform this task, a security solution uses prior knowledge of what is malicious to either place signatures on the contents of executable files and process memory or monitor the behavior of the process and decide that the behavior is malicious [2].

The behavior of a process can be defined as a series of interactions between the said application and the underlying operating system. Modern operating systems (OSs) provide several mechanisms for monitoring the behavior of processes. Security solutions typically use monitoring technologies by deploying a sensor program that uses what the OS offers and extracts higher-level knowledge from it. This higher-level knowledge is then placed in context and evaluated to decide if it is malicious or clean.

The quantity of malware is always increasing, so it is impossible for security solutions to keep up without an automatic way of learning what is malicious. This is why machine learning techniques are routinely used for malware detection [3]. Machine learning is a branch of artificial intelligence that enables computers to learn from data and make decisions or predictions without being explicitly programmed. It involves training algorithms on datasets to identify patterns and relationships, which can then be used to analyze new data [4].

For machine learning to effectively detect malware, a feature extraction, training, and detection model (algorithm) evaluation process must be established [5]. While straightforward with complete information, the challenge intensifies when the goal is to identify malicious behavior swiftly.

Firstly, it is essential to detect and block malicious activities quickly. For instance, ransomware may not be effectively identified using complete logs, as the malware itself alerts users to its presence, rendering post-infection detection redundant. If a security solution only disables the ransomware after files are encrypted, it ironically harms more than helps, as it can prevent users from paying the ransom to regain access to their files.

Secondly, accurately distinguishing malicious processes is critical. Most applications present similarly to external sensors before activating the main function and only diverge in behavior following the execution of suspicious operations.

Thirdly, clean applications might initially perform actions resembling those of malware, such as reading system information or connecting to the Internet to download and execute files, which complicates early detection efforts. Only subsequent behaviors, like displaying an installer dialog, may clarify the benign nature of the process.

Lastly, the performance impact of security solutions is a significant consideration; overly intrusive measures can disrupt organizational processes as severely as malware itself, emphasizing the need for balanced security approaches that do not undermine system usability.

Our paper proposes a potential solution to the previously described problems. We outline a methodology for training and evaluating detection models tailored for real-time malware detection. This approach involves the security solution of continuously monitoring a process throughout its execution, extracting features from the observed behavior, and assessing whether the feature vectors are malicious. This evaluation is conducted only at the optimal moments, ensuring both the effectiveness and efficiency of detection. The main contributions of our study include the following:

- We created a malware detection system that we call LEDA (Layered Event-based Malware Detection Architecture) that uses events received from the operating system to evaluate if process behavior is malicious.
- We developed a new methodology for training and evaluating machine learning models using LEDA, aimed at rapid and accurate malware identification without compromising system performance or user productivity.
- We created a new methodology and a novel algorithm to select the most suitable events that should trigger the evaluation of the machine learning detection model.
- We tested the effectiveness of our solution on a collection of new malware samples that were collected throughout a calendar year. Specifically, we assessed how training our system with data collected during the first six months impacts its performance over the last six months of the year.

The rest of this paper is structured as follows. Section 2 positions our work in the larger context of behavior-based malware detection and shows related work. Section 3 describes the technical aspects of how we built LEDA. Section 4.1 discusses how we represent monitored processes, how we use virtual machines to extract behavior, how we remove noise caused by the operating system, and how we label processes as clean or malicious. Section 4.2 shows how we train our detection model and how we automatically learn when to evaluate feature vectors. Finally, we present our experimental results in Section 5, discuss the relevance of our work in Section 6, and conclude our paper in Section 7.

## 2. Related Work

Recent studies such as [5] show that dynamic malware detection using ML algorithms can have good potential. The authors ran samples inside the Cuckoo sandbox, generated behavior reports, and converted the reports into sparse feature vectors for machine learning model training. We similarly ran samples inside a sandbox and transformed their behavior into feature vectors. We focused on detecting malware during execution, so instead of using full logs, we developed a method to evaluate malicious behavior with incomplete information. Our research used significantly more samples for tests.

Zhang et al. [6] presented a novel approach for dynamic malware detection using feature engineering and deep neural network architecture. The proposed method leverages system API calls and their arguments, utilizing feature hashing to encode these features and applying a combination of Gated-CNNs and bidirectional LSTM (Bi-LSTM) for malware detection. Information about API calls is extracted using the Cuckoo sandbox. Maniriho et al. built a similar system named API-MalDetect [7]. The authors again used the Cuckoo sandbox in an isolated dynamic analysis environment to execute the samples and extract API call sequences. They tokenized and encoded API calls into numerical representations using Keras, created dense vector representations of the API call sequences, and used deep learning to create a malware detection model. We similarly extracted information using a sandbox, but instead of API calls, we extracted higher-level events that we believe contain more semantic meaning. Translating the methods described by Zhang and Maniriho to work with our events is something that we consider to be valuable future work.

Nishiyama et al. [8] proposed using supervised classification as a baseline and changing the objective function to maximize the partial area under the ROC curve (pAUC) to result in a high true positive rate at a low false positive rate. The method was applied to proxy logs from a large enterprise network.

In [9], the authors used system log files with sequence calls recorded by IoT devices for malware detection. Random forest, XGBoost, and SVM were used for classification, and the first two obtained the best results. Mills et al. [10] showed that random forest is a resource-efficient and effective algorithm for malware detection. The authors built a real-time malware detection system that runs on affordable hardware such as a Raspberry Pi around random forests. We decided to use random forest in our experiments similarly because of the low evaluation performance impact.

Real-time malware detection during execution with incomplete logs is not an extensively studied field. At the moment of writing this article, there are currently only two approaches to the problem: using monotonic models and using time-based sliding monitoring windows, each with few exponent papers.

The authors of the [11] addressed the issue of real-time detection, noting that current classifiers rely on complete logs and are inaccurate with partial logs. Their solution involves making both feature extraction and classification monotonic, meaning that adding new log lines can only increase the likelihood of detecting malicious behavior. This approach ensures that predictions consistently become more confident over time. Predictions remain benign throughout benign files, while predictions become and stay malicious for malicious files. Our method addresses the same problems but in a different way. Instead of analyzing events as the sensors of our detection system receive them, we automatically learn for which events we should evaluate our detection model to reduce the chances of false positives and have a detector that works on partial logs.

The authors of Propedeutica [12] proposed that malware detectors use sliding windows to process and analyze system call sequences over a specific time frame or a set number of system calls to assure performance-efficient malware detection during execution. This method works on partial logs but is very vulnerable to attacks. If malicious actors know how the sliding window is created, they may simply spread malicious behavior across multiple windows. Our approach reduces performance impact by choosing when to evaluate the detection models instead. We always keep full visibility of what a process does. Furthermore, our events contain more information than a simple API call.

## 3. LEDA System Architecture

**Figure 1:**
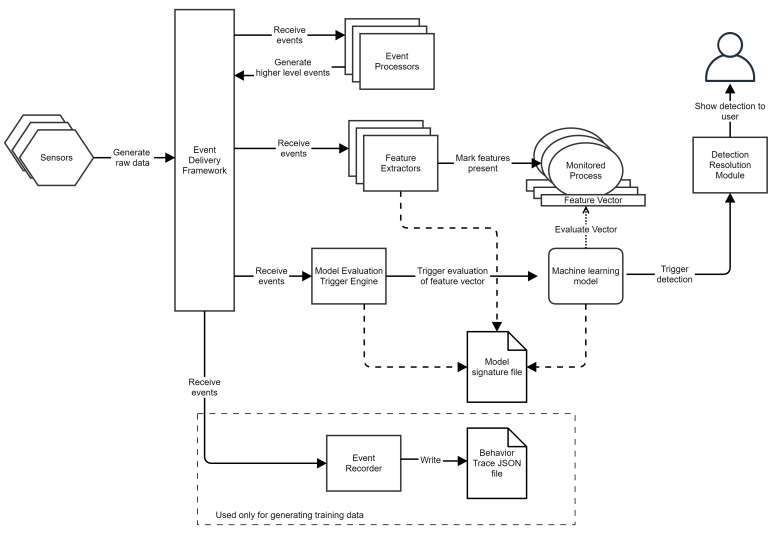
Architecture of the LEDA system.

We propose a novel detection system architecture (Figure 1) suitable for multiple operating systems we name Layered Event-based Malware Detection Architecture (LEDA). Our system collects data from an underlying operating system, processes it to extract detection-relevant information, and uses said information to decide if a monitored process is malicious or not.

LEDA is built around an event delivery framework where events generated by decoupled event producers are routed to event consumers. The event framework is a generic implementation of a message delivery system and allows components to register callbacks to be notified whenever a specific event occurs on the system. Other components may submit events to the event delivery framework. The framework allows the dispatching of events using two dispatch policies:

- Synchronous dispatch—events are submitted to event processors synchronously. This dispatch policy uses locking and extra memory allocation to dynamically allow event processors to be added and removed from the framework.
- Asynchronous dispatch—events are submitted to event processors asynchronously on a special thread pool included in the framework. Asynchronous dispatching may reduce the perceived performance impact of certain evaluations and may be required by the nature of the sensor. For example, events from the Event Tracing For Windows providers are always asynchronous on Windows.

Our system uses two different classes of events:

- Infrastructure events—They make the solution work. These events are not used for malware detection; they are internal to the system.
- Behavioral events—They express some part of the behavior of a process.

Internally, events are represented as structures containing fields that can be strings, integers, or Booleans. Each behavioral event is associated with a process. A reference to information about the monitored process is contained in the event body.

LEDA collects information from the operating system through sensors. Sensors depend on the operating system and are connected to the operating system’s facilities, which are designed for process behavior monitoring. The sensors collect data from the operating system and convert them to raw (unprocessed) events. These raw events are then submitted to the event delivery framework for dispatching to other components. Examples of sensors on the Windows operating system may include a mini-filter driver to monitor file system activity and raw disk access, kernel notification routines for processes, threads, executable images, objects, registry notifications, and event tracing for Windows consumers. On Linux, examples may include using drivers or the extended Berkeley Packet Filtering [13] facility to receive events from the operating system. On Mac OS, the Endpoint Security API [14] may be used to achieve similar results. Function hooking may supplement any missing sensor data not already offered by the OS.

Event processors consume and aggregate events from the event delivery framework. Each event processor may receive one or multiple events, generate any number of other higher-complexity events, and submit them to the event delivery framework. Event processors may store data associated with the process during aggregation and may work similarly to a state machine.

Feature extractors are a special kind of event processor. They do not generate events; instead, they evaluate each event based on a series of available predicates and generate a feature to be stored in a feature vector for the monitored process referenced in the event body.

Predicates are simple predefined operations that may be applied to fields of an event such as *isTrue*, *isFalse*, and *beginsWith*. Each field type in an event has a predefined set of predicates that logically work on the type; for example, numeric operators work only on numbers, string operators work on strings, etc.

LEDA includes multiple implementations of machine learning models that can be configured to work on feature vectors from processes. Examples of such models include random forests, Markov chains, and neural networks. A model signature file contains the parameters of the machine learning model as well as the configuration for the feature extractors that select the features for the said model.

Models are evaluated only under certain conditions. The model evaluation trigger engine decides whether to evaluate a model or not, which receives events from the event framework and applies predicates to them, similar to how feature extractors operate. Suppose the sum of predicates marks the event as a trigger for model evaluation. In that case, the model will be instructed to begin evaluation and check if a detection should be shown to the user. The configuration for the predicates for the model evaluation trigger is stored in the model signature files, along with all other parameters for the model.

A processor configuration file controls if an event processor is enabled or disabled. By updating this configuration file, an event processor may be turned off. An event processor is turned off if no other component uses the data generated by it or if some critical deployment issue may be solved by disabling the code paths triggered by the presence of that event processor. As a further optimization, the event framework internally knows how many processors are registered to receive a type of event; if an event has no listeners, event dispatching will not be started. No extra memory or computational overhead is added for events with no listeners.

As a result of our training process, we generate the model together with the logic for when to evaluate it and which event processors are active. Thus, an update to LEDA is just an update to the two configuration files.

We added a virtual “detection resolution module” to represent what a typical security solution would realize for malware detection, such as blocking the software, generating telemetry, or starting any disinfection routines.

## 4. Methodology

In this section, we describe the experimental setup and the methodologies used for detecting malware in real time through LEDA. The process involves data acquisition through malware detonation, the extraction and processing of behavioral events, and the training of machine learning models for real-time malware detection.

The following subsections provide details on the malware data collection environment, the data labeling process, the system architecture that supports event-driven detection, and the methods employed for feature extraction and model training.

### 4.1. Data Acquisition via Malware Detonation

Data are paramount for machine learning to work, especially in detecting malware during execution. Large amounts of information about execution traces are crucial for training accurate models. Currently, the only reliable method to obtain behavior traces from malicious executions is to detonate malware in sandboxes. This process involves running the malware in a controlled environment, usually a virtual machine, to observe and record its behavior without risking actual system damage. For this purpose, LEDA contains an event recorder feature that can be activated during detonation. This feature will dump all events generated for each monitored process in a behavior Trace JSON.

**Figure 2:**
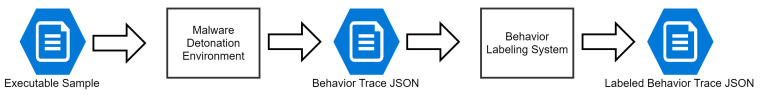
Data acquisition flow.

Figure 2 illustrates our data acquisition process. The first step for data acquisition is to run samples from a dataset within a malware detonation environment, accompanied by a modified version of LEDA, where we activate the event recorder. Our detonation environment runs on top of VMware, with multiple VMs connected to an internal network and the internet via a TOR Proxy. We modified the virtual machines to hide as many artifacts as possible that would help detect a sample that runs in a sandbox. We executed programs in the sandbox for a maximum of 5 min.

Following the detonation, the behavior Trace JSON file is extracted and sent to a behavior labeling system, which is described in more detail in the next section. This system employs an algorithm to reduce the behavior Trace JSON file to a canonical form, effectively removing noise considered irrelevant. It checks if the process is executed correctly inside the virtual machine, and finally, it checks if static signatures of anti-malware engines detect the process or artifacts it produced. After this assessment, the behavior Trace JSON is augmented with the verdict and becomes a labeled behavior Trace JSON.

#### 4.1.1. Process Behavior Representation as an Ordered Set of Layered Events

LEDA represents monitored processes as an ordered set of events generated by sensors or event processors for a given process *P*:

$$P=\{e_{0},e_{1},\ldots ,e_{n}|e_{i}\;\mathrm{is}\;\mathrm{an}\;\mathrm{event}\;\mathrm{captured}\;\mathrm{by}\;\mathrm{LEDA}\;\mathrm{for}\;\mathrm{the}\;\mathrm{process}\;\}$$

Events in *P* are always ordered by timestamp. The first event, $e_{0}$, is always the process creation event. Sensors or event processors may generate all other events. We define three layers (categories) of behavioral events based on complexity and amount of information contained:

- Layer 0: Raw sensor data—unprocessed events received from all sensors in the solution. These events answer “How did a monitored process perform an action?”
- Layer 1: Filtered and expanded data—events resulted from the processing of layer 0 events by event processors. These events aim to infer “What did a monitored process do on a higher level?’’
- Layer 2: Context—these events show the impact of the malware on the system. They show more complex, meaningful behavior extracted by event processors from Layer 0 and Layer 1 events. These events are used to infer “Why a sample performs a given action?’’

Consider the following example that will clarify the separation in layers. Assume that a monitored process would like to copy a file to the startup folder on Windows. This security-relevant event would be translated to a Layer 3 kind of event, for example, FILE_COPY_TO_STARTUP_FOLDER. To achieve this, a monitored process may perform any of the following:

- Call CopyFile* Windows API.
- Copy in chunks of an existing file using file reads and writes.
- Use the COM object IFileOperation API CopyItem to copy the file.

Some event processors may generate a simple FILE_COPY event in each case, A, B, or C. This event is considered at Layer 1 because it shows what happened after the call to CopyFile*, a succession of reads and writes, or a COM call to IfileOperation::CopyItem.

In each case, A, B, or C, sensors need to generate one or more Layer 0 events that will be submitted to processors at level 1 and allow them to generate the FILE_COPY event. For case A, the call to CopyFile needs to be intercepted and submitted to a processor, maybe in the form of API_CALL_COPY_FILE. For case B, multiple FILE_READ and FILE_WRITE events and possibly a FILE_CLOSE event may be aggregated in such a way as to determine if the written file was identical to the source file. For case C, more complicated logic to interpret the COM call for IFileOperation and, if the COM object was created as part of a local server, to find the corresponding dllhost.exe surrogate that will perform the action is required. This logic will be based on multiple raw sensor data.

The Level 1 FILE_COPY event will be further evaluated by Layer 2 processors to determine if the behavior is security relevant and, if so, to generate a new event. For our example, because the destination of the copy is the Windows startup folder, a FILE_COPY_TO_STARTUP_FOLDER event will be generated.

Processes for cases A, B, and C will have the events at all levels in their representation; FILE_COPY and FILE_COPY_TO_STARTUP_FOLDER will be common. Still, events before them in the representation will be different and correspond to how the copy was performed.

Our previous example hints toward the complexity of event processors. These processors extract security-relevant information about process behavior, seen as a set of actions that have lasting effects on the operating system [15] based on sensor data. Examples of actions are changes affecting the file system or the Windows registry, a process injecting code into another process, creating and using synchronization primitives, network activity, or creating, starting, and stopping OS services. A subset of security-relevant actions that a Windows process may perform is given in Table 1. We assign relevance to various actions based on guidelines described in the widely used in cybersecurity field MITRE ATT&CK matrix [16]. Each tactic and sub-tactic described in the matrix has one or more processors that generate suitable higher-level events for it.

Internal implementations of event processors differ from case to case based on the lower-level events they consume and the higher-level events they generate. In some cases, a processor may simply need to check if a file has a specific extension (for example, .cpl); in other cases, processors might check if a file write compromises the file header of a well-known media file or may contain complex state machines that correlate multiple file creations to conclude that a ransom note was dropped in folders that contained documents.

#### 4.1.2. Environment-Specific Data

All events contain machine-specific information. The startup folder is a good example of this because on Windows; it is located in a user directory, specifically as a subdirectory of %APPDATA%, which resolves to C:\Users\<Username>\AppData\Roaming.

This machine-specific information may hinder malware detectors trained on process information extracted by LEDA. We define a NORMALIZE function for each event that replaces machine-specific data with standardized data, for our example, NORMALIZE(C:\Users\anonymized_user\AppData\Roaming) = C:\Users\<Username>\AppData\ Roaming. The same process applies to process ids and other data that are machine and execution dependent.

Our NORMALIZE function is applied to each machine that has LEDA installed. For this to work, LEDA needs to know machine-specific data such as user names and special user folders. Some folders, such as “Program Files’’ or “Windows’’ can be derived easily. User folders, on the other hand, require more complex logic. LEDA monitors each user login and leverages the registry to obtain the information required. For example, the registry key Registry<\USER_SID>\Software\Microsoft\Windows\CurrentVersion\ Explorer \User Shell Folders stores user-specific folder paths within Windows. The <USER_SID> refers to the unique *Security Identifier (SID)* for each user account, ensuring that settings are isolated per user. Under the User Shell Folders subkey, the operating system defines the locations for various special folders, such as “Desktop’’, “Documents’’, and “Downloads”. In the rest of the paper, we define $e^{\prime }$ as the result of applying NORMALIZE to all e fields and changing their content with the result. This allows us to introduce the notion of a normalized process representation $P^{\prime }$ defined as

$$P^{\prime }=\left\{e^{\prime }|e^{\prime }=\mathrm{NORMALIZE}\left(e\right),\;e\in P\right\}$$

#### 4.1.3. Noise in Event Representation

Processes, be they malicious or clean, run on top of an operating system and use various code libraries to perform operations that help them achieve the purposes for which they were created. The operating system has various operations it needs to perform in the context of a process to allow it to start executing. For example, on Windows, most processes start their execution by mapping the main executable image, mapping ntdll.dll, kernel32.dll, and kernelbase.dll; following that, a sensor may see csrss.exe writing the process memory of the new process or even various wmiprvse.exe processes reading and writing the virtual memory for the said process. This is all normal for the operating system to do, so for malware detection, this represents noise.

One of the key challenges we faced when building LEDA was reducing this noise, especially in the training process, to avoid training our malware detector to trigger on standard operating system operations. This problem becomes even more complicated because operating system updates may introduce more noise or change previous noise events. We introduce the notion of a demonized process to be

$$\hat{P}=P^{\prime }-Noise$$

The subtraction operation involves identifying and excluding non-security-related events, which are introduced by the operating system for all processes, during feature extraction.

We aim to automatically compute Noise for each operating system version on which LEDA runs.

Because we want to detect malware during execution, another key challenge is to check if the malware actually worked inside the virtual machine. A program marked as malicious by static signatures may require components not present on the machine or a connection to a command and control server that was rightfully taken down before it starts showing the malicious behavior. Malicious samples may detect the virtual environment; they may crash before performing malicious operations or after showing some part of the malicious behavior. Behavior extracted for malfunctioning samples may consist only of noise generated, for example, by how the operating system handles a crash.

For a given operating system, we can check what kind of events LEDA generates for various cases in which samples malfunction by creating simple probe programs that show the malfunction. We know what kind of programs to create by detonating malware, automatically and manually checking logs, and reproducing the issues. For this study, we created a large number of programs in various programming languages that each replicated a potential issue. We refer to these programs as calibration programs. Examples of the calibration programs include the following:

- Programs that connect to the Internet and wait for commands;
- Programs that depend on certain files to be present on the disk (such as an external module that should be found in the same folder as the program);
- Programs that crash and trigger Windows Error Reporting;
- Programs that check if they are running inside a virtual machine and stop if they detect one.

The calibration programs needed to check if the malware executes successfully can help us compute noise because the events generated by the OS to ensure processes work are common for all processes.

For any operating system update performed on virtual machines in our environment, we used Algorithm 1 to compute each calibration program’s noise and the denoised event representation. We refer to the denoised representation of a calibration program as a known behavior profile, and the name of all the profiles as BehaviourProfiles.
**Algorithm 1** Machine Calibration 1:Calibration = new Set 2:**for** each calibration program *c* **do** 3:      detonate *c* in a virtual machine with LEDA 4:      $C_{i}$ = ordered set of events seen by LEDA 5:      $C_{i}^{\prime }=\mathrm{NORMALIZE}\left(C_{i}\right)$ 6:      insert $C_{i}^{\prime }$ in Calibration 7:**end for** 8:$Noise={⋂}_{C^{\prime }\in \mathrm{Calibration}}C^{\prime }$ 9:behaviourProfiles10:**for** each $C^{\prime }$ in Calibration **do**11:      $\hat{C}=C^{\prime }-Noise$12:      insert $\hat{C}$ in BehaviourProfiles13:**end for**14:**return** Noise, BehaviourProfiles

#### 4.1.4. Behavior Labeling

We label the behavior of processes using Algorithm 2. First, we check if executing the sample results in a known behavior profile (i.e., the behavior corresponds to a known calibration program after removing the noise). Second, we check if the sample or an indicator of compromise (IOC) displayed during detonation corresponds to a known malware family. We extract IOCs from a process by looking at the main image of the process, all additional files created by it, and all URLs the process interacts with.

We computed labels for IOCs using a modified version of [17]. Our version of the algorithm takes into account that security solutions often buy signatures from other independent vendors. We also used a non-public cleanset to avoid false positives, found on VirusTotal. We present our logic in Algorithm 3. Our algorithm uses three configuration dictionaries. The DETECTION_TYPES dictionary contains the anti-malware engine and malware type names such as “CoinMiner’’, “Trojan’’, “GenericKD’’, “Win32’’, “Variant’’, “Ransom’’, “SoftwareBundler’’, or “AdWare”. The FALSE_POSITIVE_PRONE_TYPES contains detection names that we consider to be very prone to false positives, such as “Heuer’’, “Generic’’, “AIDetect’’, “Suspicious’’, or “PossibleThreat”. Our preliminary tests showed that these kinds of tags correspond to badly tuned detections with many false positives, so we ignored them. Finally, our algorithm uses a table of aliases for malware types, which we named MALWARE_TYPE_ALIASES. Listing 1 shows an example of such a table.
**Algorithm 2** Behavior Labeling**Require:** $\hat{P}$—denoised event form of process P 1:**for** each $\hat{B}$ in BehaviourProfiles **do** 2:      **if** $\hat{P}=\hat{B}$ **then** 3:          **return** label of $\hat{B}$ 4:      **end if** 5:**end for** 6:IOC_LIST = new List 7:**for** each *e* in $\hat{P}$ **do** 8:      **for** each URL or file IOC in *e* **do** 9:          insert IOC in Calibration10:      **end for**11:**end for**12:**for** each ioc in IOC_LIST **do** Label = COMPUTE_LABEL(ioc)13:      **if** Label != “CLEAN” **then return** Label14:      **end if**15:**end for**16:**return** “CLEAN”
**Listing 1.** Malware type aliases.“trojan”: [“trojan”, “tr/”, “troj”], “ransomware”: [“ransomware”, “ransom”, “ranserkd”, “filecoder”, “ransom_blocker”, “ransomx”], “sof twarebundler”: [“sof twarebundler”], “adware”: [“adware” ], “backdoor”: [“backdoor”, “remoteadmin”], “keylogger”: [“keylogger” ], “pua”: [“application”, “dangerousobject”, “unsafe”, “riskware”, “Program”, “Hack”, “GameHack”], “patcher”: [“patcher”], “dropper”: [“downlader”, “drop”], “miner”: [“miner”]

Algorithm 3 is structured into four main parts.

First, the algorithm queries the non-public cleanset to check if the IOC (file hash or URL) is listed there. If the cleanset identifies the IOC as “CLEAN’’, no further processing is needed, and the algorithm returns “CLEAN’’ immediately.

If the IOC is not found in the cleanset, the algorithm proceeds to query VirusTotal. VirusTotal provides detection results from multiple anti-malware engines, which are stored in *VIRUS_TOTAL_LABELS*. These labels are then processed to remove any duplicates, ensuring that the algorithm works with unique information.

Next, the algorithm processes the retrieved labels. This involves normalizing the labels and breaking them into relevant components to identify key attributes, such as malware family names. To reduce false positives, the algorithm excludes detections from engines with names listed in *FALSE_POSITIVE_PRONE_TYPES* or components that are too generic or consist primarily of hexadecimal digits.

Finally, the algorithm determines the malware family and type for the IOC. It counts the frequency of family names within the processed labels and matches them against the *MALWARE_TYPE_ALIASES* table. The family with the highest occurrence is selected as the most likely family. Similarly, the algorithm identifies the most likely type based on the matches with the aliases, and the two values are combined to form the final label for the IOC.

**Algorithm 3** IOC Labeling**Require:** IOC—as file hash or URL  1:result = QUERY_CLEANSET(IOC)  2:**if** result = “CLEAN’’ **then**  3:      **return** “CLEAN’’  4:**end if**  5:VIRUS_TOTAL_LABELS = QUERY_VIRUS_TOTAL(IOC)  6:UNIQUE_LABELS = REMOVE_DUPLICATES(VIRUS_TOTAL_LABELS)  7:FAMILY_APPEARANCES = new Dictionary(String, Int)  8:**for** label in UNIQUE_LABELS **do**  9:      normalizedLabel = label.replace(“:[]/()! -:’’, “.’’) 10:     **for** component in normalizedLabel.split(“.’’) **do** 11:           **if** component is already in FAMILY_APPEARANCES **then** 12:                 FAMILY_APPEARANCES[component] += 1 13:                 **continue** 14:          **end if** 15:          **if** len(component) < 4 **then** 16:               **continue** 17:          **end if** 18:          **if** component in DETECTION_TYPES **then** 19:               **continue** 20:          **end if** 21:          **if** component in FALSE_POSITIVE_PRONE_TYPES **then** 22:             **continue** 23:          **end if** 24:          **if** component contains mostly hex digits **then** 25:               **continue** 26:          **end if** 27:          FAMILY_APPEARANCES[component] = 0 28:      **end for** 29:**end for** 30:**if** FAMILY_APPEARANCES is empty **then** 31:      **return** “CLEAN’’ 32:**end if** 33:Family = max(FAMILY_APPEARANCES) 34:Type = “Generic’’ 35:TYPE_APPEARANCES = new Dictionary(String, Int) 36:**for** label in UNIQUE_LABELS **do** 37:      **for** type in MALWARE_TYPE_ALIASES **do** 38:          **for** alias in MALWARE_TYPE_ALIASES[type] **do** 39:             **if** alias in label **then**: 40:                 **if** type not in TYPE_APPEARANCES **then** 41:                     TYPE_APPEARANCES[type] = 0 42:                 **end if** 43:                 TYPE_APPEARANCES[type] += 1 44:             **end if** 45:          **end for** 46:      **end for** 47:**end for** 48:**if** len(TYPE_APPEARANCES) > 0 **then** 49:      Type = max(TYPE_APPEARANCES) 50:**end if** 51:**return** Type + “.’’ + Family


### 4.2. Model Training

LEDA detects malware by evaluating feature vectors assigned to processes using machine learning models. Evaluation is carried out only on specific trigger events. During training, we need to learn the model together with the optimal triggers for it.

We start the training process by detonating malware and cleaning applications in virtual machines. For each detonated sample *S*, we compute the normalized event form $S^{\prime }$ and the denoised form $\hat{S}$. We use all implemented feature extractors on $\hat{S}$ and extract $F(\left(\hat{S}\right))$, the complete feature vector of sample *S*. At this point, we remove samples labeled as having a known behavior profile (i.e., similar to samples in behaviourProfiles) because we know they malfunctioned.

As a result of this first step, we know the features $F(\left(\hat{S}\right))$ that can be extracted for all samples and can label samples as Malicious and Clean. What is left is to compute F(B) for any known B∈behaviourProfiles and label these feature vectors as Clean so that our models can learn that malfunctioning samples are not malicious.

The reunion of all $F(\left(\hat{S}\right))$ for our samples results in a huge number of distinct features that make the remainder of the training process impractical. We resolve this issue using a feature selection algorithm on $F(\left(\hat{S}\right))$ for all samples *S*. For this purpose, we use a simple Logistic Regression with L1 regularization to select a fixed number of the most relevant features. We relabel the selected features to be consecutive and generate the configuration data for the new model’s feature extractors to generate only the relevant features and store them in the model configuration file.

After feature selection, for each detonated sample *S*, we can compute $F^{\prime }\left(\left(\hat{S}\right)\right)$, the feature vector that contains only features that were considered relevant. We can then split data into training and testing data and proceed with training a detection model. We store the parameters of this model in the corresponding configuration file.

What is left is to select the optimal list of trigger points to check if the feature vector for a process is malicious or clean. This list needs to contain as few triggers as possible to reduce the computational overhead and should make LEDA detection metrics as good as possible.

Typical detection metrics for machine learning models are accuracy, precision, recall, and F1 Score. Accuracy measures the proportion of correctly classified samples (both malware and benign) out of the total number of samples. High accuracy indicates that the model correctly classifies most of the samples; however, if the dataset used for training is imbalanced (e.g., the training set contains significantly more malware than clean samples), this metric is misleading. Precision measures the proportion of correctly identified malware instances out of all instances classified as malware. High precision indicates that the model rarely classifies benign samples as malware, which is important in reducing false alarms. Recall measures the proportion of correctly identified malware instances out of all actual malware instances. High recall indicates that the model identifies most of the actual malware samples. Finally, the F1 Score is the harmonic mean of precision and recall. When we select the trigger points, we consider the F1 Score to be the metric to be optimized because it provides a single metric that balances both concerns of false negatives and false positives.

LEDA detects malicious processes during execution. This means that the denoised representation of events generated for a given process $\hat{P}$ changes every time *P* performs a new action. This directly implies that the feature vector $F^{\prime }\left(P\right)$ also changes at the same rate and that the feature vector $F^{\prime }\left(P\right)$ for process *P* is different at each evaluation trigger point.

**Figure 3:**
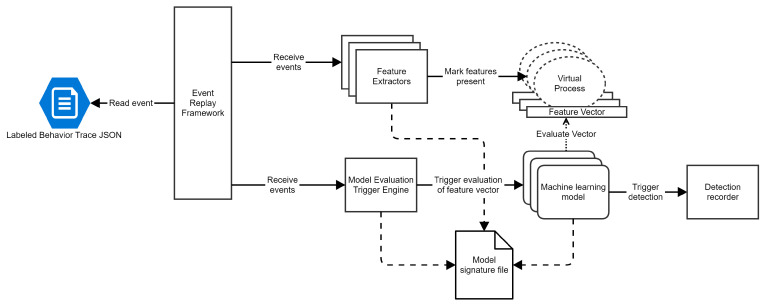
Simplified LEDA architecture for model prototyping.

Selecting the correct trigger points requires us to know how the detection model will label the feature vectors for each trigger point. To achieve this, we utilize a model prototyping system that contains only the feature extractors, the trigger engine, and the machine learning model. The architecture of this system is presented in Figure 3. The event framework is replaced with an event replay framework that reads events from the behavior Trace JSON and sends them to the feature extractors.

Using the prototyping system, we can define triggerPrediction[N][M], a matrix, where *N* is the number of samples, *M* is the number of triggers, and triggerPrediction[S][T] = the label given by the model when evaluating feature vector $F^{\prime }\left(S\right)$ at moment *T*. For simplicity, we consider labels 0 to represent “CLEAN’’ and 1 otherwise.

We now need to select a set of triggers TS, such as the F1 Score being optimal for them. We consider the label generated by the model for a sample *S* and trigger set TS to be 0—“CLEAN’’—if triggerPrediction[S][T] is 0 for all triggers *T* in TS. Theoretically, the best set of triggers will have the highest F1 Score; however, in practice, because the total possible combination of triggers is a very large number, we can consider the first set of triggers that has an F1 Score larger than a predefined threshold. Empirically, we observed that lower thresholds represent fewer triggers, which improves performance.

At this point, the trigger selection algorithm becomes simple. We generate all trigger sets of one trigger, then two triggers, and so on until we find a subset that has an F1 Score larger than the threshold. Although computationally intensive, this problem can easily be parallelized, thus making training times reasonable if the threshold is permissive. We empirically selected a 0.9 F1 Score to be good enough.

As a final step, we store the configuration for the found triggers in the model configuration file, and training is carried out.

## 5. Tests and Results

### 5.1. Dataset Presentation

We tested the solution using a dataset consisting of 70,000 known malicious samples and 13,000 known clean samples that can be executed successfully on Windows 10. We collected these samples during 2018 via voluntary sample submission, malware shares, and honeypots. We grouped samples per month based on the time we inserted the sample in our internal database.

We split our dataset into four categories:

- Documents → macro-enabled documents that can be executed using Microsoft Office products like Word, Excel, or PowerPoint.
- Scripts → various PowerShell, JavaScript, or visual basic scripts that are executed with a script interpreter.
- Installers → various executables created using some installer framework (examples include MSI, InstallShield, etc.).
- Executabl → Various malicious windows PE files created for x64 or x86 architecture.

All samples in the collection function correctly. They do not crash and do not perform evasion in our detonation environment. They perform at least one action, which we consider to be an indicator of maliciousness. All samples were labeled using static signatures, as described in Algorithm 2. We included both installers of an application and the applications installed for clean samples. We also included various scripts used for system administration that are clean and macro-enabled documents that are not malicious. Table 2 provides a breakdown of the dataset.

We detonated documents with macros with macros set to execute by default without requiring user interaction.

We split the dataset into multiple sub-datasets for the following tests based on collection dates. A summary of the splits for clean samples may be seen in Table 3.

We added an extra metric for malicious samples to show the distribution of most spread malware families over a year. For this metric, we selected the top 50 malware families out of the 137 families we identified Jan-Jun. We measured the top families on each validation period to check how malware changes in the market. The malicious sample distribution is available in Table 4.

A summary of how the members of the most spread 50 families changed in the market during 2018 can be seen in Table 5.

### 5.2. Measurement of Changes in Features and Trigger Points Based on Different Training Sets

In this experiment, we wanted to see what would happen to the selected features and triggers if we retrained on different kinds of malware. Based on L1 regularization, we selected the top 10,000 features for this experiment. We let the trigger selection algorithm select the number and types of trigger points.

#### 5.2.1. Checking Stability of Features and Trigger Points as Time Passes

First, we checked if retraining on different datasets during a year changes the trigger points or the relevant features selected in the top 10,000 features. We created a baseline by training on the data from January to June and then compared four batches of retrainings for pairs of 1000 samples for every two months. We also conducted validation on 10,000 random data from January to June to check what changed if we reduced the number of samples we trained on. The results can be seen in Table 6.

The findings indicate that training on smaller data results in fewer trigger points that are deduced as relevant by the system. However, trigger points maintain their relevance throughout the year. Training on data from the first half of the year captures most of the necessary trigger points for evaluations, with only samples from November to December leading to the identification of new ones. Additionally, training on sufficiently large datasets results in selecting relevant and stable features that remain consistent throughout the year.

#### 5.2.2. Checking Stability of Features and Trigger Points Based on Different Types of Infection Media

Secondly, we wanted to see how trigger points and features would change if we trained only on specific application classes. We created a baseline by training on all training sets from January to June. After this, we grouped the remainder of the samples into categories and trained only in those categories. The results of the experiment can be seen in Table 7.

The results indicate that training exclusively on a specific category of samples, such as documents, scripts, or installers, leads to significantly different outcomes in the features that the models consider. Conversely, training on larger and more varied datasets results in selecting robust trigger points relevant across multiple categories of executables.

### 5.3. Detection Evaluation of the Model

We wanted to measure the system’s detection performance for the second experiment. We trained the system on the data from January to June and tested on validation datasets. We used a simple random forest model to test our methodology. The model we used is equivalent to scikit-learn RandomForestClassifier(n_estimators=100,oob_score=True,n_jobs=−1) [18,19]. We measured the standard parameters, precision, recall, accuracy, and F1 Score.

#### 5.3.1. Multi-Point Evaluation Based Only on Learned Triggers

For the first experiment, we trained models on the datasets from January to June and validated them on the validation datasets. The results can be seen in Table 8.

The table’s results conclude that the system produces usable results with more than 0.9 accuracy· Moreover, the F1 score decreases over time. This is in line with the changes in malware distribution in the market and can be counteracted with the deployment of new, updated models.

#### 5.3.2. Adding Evaluation with Full Logs to the System (a Forced Trigger on the Process End)

The previous experiments proved that some changes might occur to the trigger points needed over time. Therefore, we decided to test what happens if we always trigger an evaluation on process termination to access full logs. We showcase the results in Table 9.

The results show that the evaluation metrics are better with the addition of full log evaluation. This shows that the features are correctly learned, and it also might show that some samples are vastly different than the majority of samples in the dataset, so they require some other evaluation trigger.

### 5.4. Performance Evaluation of the System

We tested LEDA’s performance impact on a system with an Intel Core i7-11700 @ 2.50 GHz, 32 GB of RAM, and an NVMe SK hynx 512 GB disk running Windows 11. Before running any test, we configured the machine to have as few interfering processes as possible; for example, we disabled all updates, stopped all non-important services, and disabled Windows Defender.

We used two sets of tests: micro-benchmarks that measure individual operation overheads (similar to the ones described by AV-Test [20]) and PCMARK [21] scores. We tested LEDA and LEDA with a forced evaluation on process termination against the system without a security solution.

**Figure 4:**
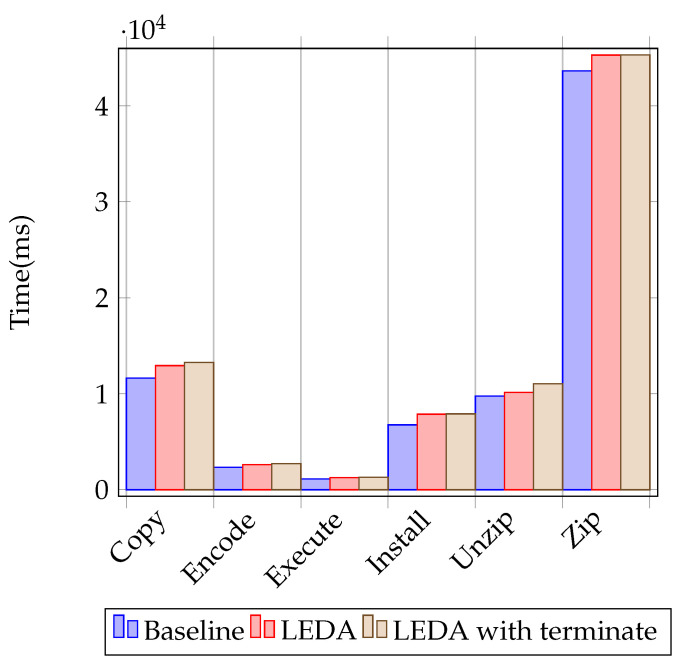
Performance test results for user activity benchmarks (lower is better).

For the micro-benchmark tests, we ran each micro-benchmark a total of 12 times, removed the highest two and lowest results, and created an average for the eight remaining results. The error margin for the test was ±0.35%. We display the result averages in Table 10 and Figure 4. Comparing the results, we see that LEDA introduces a small performance impact, even with added triggers on process termination.

**Figure 5:**
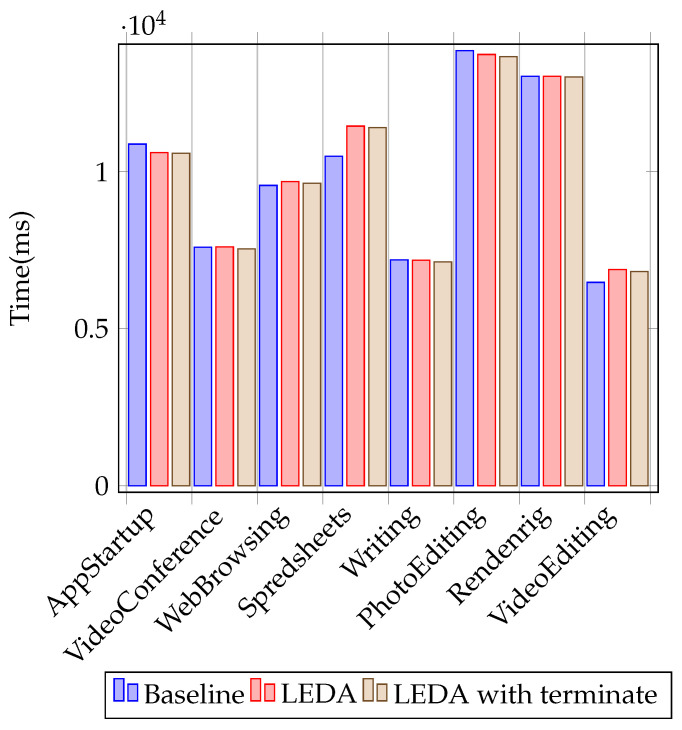
PCMARK 10 test results (higher is better).

We confirmed our results using PCMARK 10, a widely used system benchmark that assigns performance scores to various operations that a user may perform. Higher scores represent better performance. The average results for running PCMARK 10 are shown in Table 11 and Figure 5. The performance impact of LEDA is again small, even with the addition of an evaluation point on process termination.

## 6. Discussion

In our experiments, we used 70,000 known malicious entries (5000 documents, 2000 scripts, 8000 installers, and 55,000 files from other categories) and 13,000 clean samples (1000 documents, 1000 scripts, 1500 installers, and 9500 other categories) collected during 2018. We trained on samples from the first part of the year (January to June) and tested the temporal decay in the detection performance of the model by looking at the F1 Score for the validation set from the first 6 months (January to June), then on new samples in 2 month periods (July–August, September–October, and November–December). The F1 Score decays starting from 0.9349, to 0.9088, to 0.9030, and finally 0.8913. Detection can be improved by also looking at the full logs (i.e., when processes terminate), with the F1 Score going from 0.9415 to 0.9319, to 0.9243, and finally to 0.9135.

Comparing our work with other papers is difficult. Studies such as [3] recognize dataset issues as the most common research gap in the selected studies. There is currently no standard set of malicious programs that one can execute in a sandbox to obtain behavior information. We present the F1 scores for related work here, but please note that the authors used different test datasets and did not perform tests to see how the model decays over time.

The authors of [5] achieved a perfect F1 Score, 100% accuracy, 100% precision, and 100% recall. The number of samples they used was very small (373 samples), and they performed no real-world tests with malware collected over larger periods.

In [10], the authors traind a random forest that has a high F1 Score of one, but they recognized that the small dataset (146 malware samples) was the main limitation of their work.

API-MalDetect [7] uses more data than the previous two works. The authors used 8351 samples (with 4787 benign and 3564 malware) for training. In the experiments, testset 1 had 1343 samples with 90% and 10% representing benign and malicious samples; testset 2 had 1511 samples where 80% represented benign samples, while 20% represented malware; and finally, testset 3 had 1343 samples with 90% clean and 10% malware samples. The authors achieve F1 Scores of 0.9681 on testset 1, 0.9856 on testset 2, and 0.9537 on testset 3.

Propedeutica [12] achieves a 0.9724 F1 Score using a dataset of 9115 malicious programs and 1338 clean samples.

Another interesting observation that can be extracted from our data is that training models only on a category of samples, like documents, scripts, or installers, produces radically different results from the features that these specified models consider relevant. This suggests that specialized models are needed for different kinds of malware delivery mediums.

Our study used a simple random forest that worked on features using simple predicates. As future work, we consider using encoders to extract features from LEDA events, using deep learning to create more complex models, and finally, training and testing on even larger datasets.

## 7. Conclusions

Our paper presented LEDA, an event-driven architecture for malware detection during execution. This system is based on incomplete logs that continuously update with process activity as soon as sensors detect processes performing various actions. LEDA sees processes as a list of events on which it can apply feature extractors to create feature vectors for processes and evaluate these feature vectors with previously trained malware detection models.

Another key contribution to malware detection is Algorithm 1, which can identify noise introduced by the operating system during program execution. We can remove this noise by training machine learning models on only relevant data.

Our final major contribution is a methodology for determining the optimal timing for evaluating machine learning models. Using our proposed approach, we can create a malware detection system that works on incomplete logs without affecting system performance.

We tested LEDA using malware samples from 2018. We separated the malware based on the month it was first added to our database and measured the temporal decay in our system’s F1 Score during the year, achieving, in our best case, a decay starting from an F1 Score of 0.9415 close to the training time and finishing with an F1 Score of 0.9135 at the end of the year.

Finally, we tested LEDA’s performance impact using industry-specific benchmarks and showed that it is very small.

We believe that real-time malware detection based on incomplete logs is a very important research subject that is seldom addressed in the literature, and we hope that LEDA can inspire other researchers to work on it.

## Figures and Tables

**Table 1:** Subset of security-relevant actions that a Windows process may perform.

Event Class	Action Type	Description
Code Injection	Write Memory	Through various methods, a process executes code in the context of another process.
	Create Remote Thread	
	Change Execution Context	
	Load DLL	
Event Hooks	Registering an Event Hook	Similar to code injection, but for all processes instead of a specific target process.
Services	Install/Change Service	A Windows service or a driver is installed on the machine. Persistence is assured, and system-level access may be obtained.
	Install/Change Driver	A driver is installed or changed, which may lead to persistence and system-level access.
Persistence	Writes to predefined registry locations	Process will restart after a reboot. This often involves modifications to registry keys.
	WMI event subscription	Process uses WMI to create persistent event subscriptions that trigger actions after a reboot.
Process Termination	Specific API	A running process is terminated.
	Handle Exhaustion	
	Termination message	
	ALPC port message	
	APC scheduling	
Process Creation	Start a new process	A new process is created.
Win32 Desktops	Create a new desktop	The default desktop is changed or a new desktop is created.
	Change the current desktop	
Inter-process Communication	Dynamic Data Exchange (DDE)	Application uses existing OS features to execute actions out-of-process.
	Component Object Model (COM)	
	Remote Procedure Call (RPC)	
	Local Procedure Call (LPC)	
	Advanced LPC (ALPC)	
	Window Messages	
Network Communication	[multiple]	Process uses network communication features.
Direct resource access	Network	Process bypasses the common OS activity monitoring facilities by accessing the hardware directly.
	Disk	
	RAM	

**Table 2:** Sample sizes used for testing.

		Documents	Scripts	Installer Type	Executables (Other Type)
Known malicious entries	70,000	5000	2000	8000	55,000
Clean samples	13,000	1000	1000	1500	9500

**Table 3:** Clean sample distribution (based on collection dates).

Time Period	Clean Samples	Documents	Scripts	Installers	Executables
Jan–Jun (training)	9000	400	400	900	7300
Jan–Jun (validation)	1000	150	150	150	550
Jul–Aug (validation)	1000	150	150	150	550
Sep–Oct (validation)	1000	150	150	150	550
Nov–Dec (validation)	1000	150	150	150	550

**Table 4:** Malicious sample distribution (based on collection dates).

Time Period	Malicious Samples	Number of Families	Documents	Scripts	Installers	Executables	Malware without a Know Family	Malware in the Selected 50 Families	Malware in Other Families	Changes to Original Top 50 Families
Jan–Jun (training)	30,000	137	3000	1400	4000	21,600	4429	23,430	2141	0
Jan–Jun (validation)	10,000	89	500	150	1000	8350	1261	7747	992	6
Jul–Aug (validation)	10,000	104	500	150	1000	8350	1527	6128	2345	6
Sep–Oct (validation)	10,000	92	500	150	1000	8350	1499	5941	2560	8
Nov–Dec (validation)	10,000	93	500	150	1000	8350	1367	5689	2944	10

**Table 5:** Family distribution percentages.

Top Family Distributions	Jan–Jun (Training)	Jan–Jun (Validation)	Jul–Aug (Validation)	Sep–Oct (Validation)	Nov–Dec (Validation)
In top 50 families (percent)	78.1	77.47	61.28	59.41	56.89
Other (percent)	21.9	61.28	38.72	40.59	43.11

**Table 6:** Stability of features and trigger points as time passes.

	Malicious Samples	Clean Samples	Number of Trigger Points	New trigger Points vs. Jan–Jun	Different Features vs. Original Set
Original training set (Jan–Jun)	30,000	9000	43	0	0
Jan–Jun (validation)	10,000	1000	34	0	12
Jul–Aug (validation)	10,000	1000	33	0	14
Sep–Oct (validation)	10,000	1000	35	0	16
Nov–Dec (validation)	10,000	1000	34	1	14

**Table 7:** Stability of features and trigger points based on types of infection media.

	Malicious Samples	Clean Samples	Number of Trigger Points	New Trigger Points vs. Original Set	Different Features vs. Original Set
Original training set (Jan–Jun)	30,000	9000	43	0	0
Documents	5000	1000	18	2	1387
Scripts	2000	1000	24	0	2533
Installers	8000	1500	25	1	1034
Executables (other)	55,000	9500	39	0	47

**Table 8:** Detection results based on learned triggers.

	Malicious Samples	Clean Samples	TP	TN	FP	FN	Precision	Recall	Accuracy	F1 Score
Jan–Jun (validation)	10,000	1000	9649	993	7	351	0.9067	0.9649	0.9675	0.9349
Jul–Aug (validation)	10,000	1000	9157	995	5	843	0.902	0.9157	0.9229	0.9088
Sep–Oct (validation)	10,000	1000	9049	992	8	951	0.9012	0.9049	0.9128	0.903
Nov–Dec (validation)	10,000	1000	8842	998	2	1158	0.8986	0.8842	0.8945	0.8913

**Table 9:** Detection results based on full logs.

	Malicious Samples	Clean Samples	TP	TN	FP	FN	Precision	Recall	Accuracy	F1 Score
Jan–Jun (validation)	10,000	1000	9777	993	7	223	0.9078	0.9777	0.9791	0.9415
Jul–Aug (validation)	10,000	1000	9588	989	11	412	0.9065	0.9588	0.9615	0.9319
Sep–Oct (validation)	10,000	1000	9439	984	16	561	0.9056	0.9439	0.9475	0.9243
Nov–Dec (validation)	10,000	1000	9242	992	8	758	0.9031	0.9242	0.9304	0.9135

**Table 10:** Test results for user activity benchmarks (lower scores are better).

	Baseline	LEDA	LEDA with Terminate
copy files	11,638	12,932	13,262
encode	2328	2617	2709
execute	1125	1255	1284
install	6758	7866	7904
unzip	9751	10,127	11,043
zip	43,639	45,291	45,305

**Table 11:** PCMARK 10 test results (higher scores are better).

	Baseline	LEDA	LEDA with Terminate
AppStartup	10,871	10,603	10,583
VideoConference	7592	7603	7535
WebBrowsing	9558	9683	9626
Spredsheets	10,483	11,444	11,395
Writing	7189	7181	7127
PhotoEditing	13,847	13,726	13,656
Rendenrig	13,031	13,027	13,009
VideoEditing	6474	6878	6815

## Data Availability

The authors do not have permission to disclose the data.
